# A cluster randomized controlled trial of a clinical pathway for hospital treatment of heart failure: study design and population

**DOI:** 10.1186/1472-6963-7-179

**Published:** 2007-11-07

**Authors:** Massimiliano Panella, Sara Marchisio, Andrea Gardini, Francesco Di Stanislao

**Affiliations:** 1 Department of Clinical and Experimental Medicine, University of Eastern Piedmont "A. Avogadro", Novara, Italy; 2 Department of Hygiene and Public Health, University "Politecnica delle Marche", Ancona, Italy; 3 Unit for Quality Improvement, Regional Healthcare Agency of Marche, Ancona, Italy

## Abstract

**Background:**

The hospital treatment of heart failure frequently does not follow published guidelines, potentially contributing to the high morbidity, mortality and economic cost of this disorder. Consequently the development of clinical pathways has the potential to reduce the current variability in care, enhance guideline adherence, and improve outcomes for patients. Despite enthusiasm and diffusion, the widespread acceptance of clinical pathways remain questionable because very little prospective controlled data demonstrated their effectiveness. The Experimental Prospective Study on the Effectiveness and Efficiency of the Implementation of Clinical Pathways was designed in order to conduct a rigorous evaluation of clinical pathways in hospital treatment of acute heart failure. The primary objective of the trial was to evaluate the effectiveness of the implementation of clinical pathways for hospital treatment of heart failure in Italian hospitals.

**Methods/design:**

Two-arm, cluster-randomized trial. 14 community hospitals were randomized either to arm 1 (clinical pathway: appropriate use of practice guidelines and supplies of drugs and ancillary services, new organization and procedures, patient education, etc.) or to arm 2 (no intervention, usual care). 424 patients sample (212 in each group), 80% of power at the 5% significance level (two-sided). The primary outcome measure is in-hospital mortality. We will also analyze the impact of the clinical pathways comparing the length and the appropriateness of the stay, the rate of unscheduled readmissions, the customers' satisfaction and the costs treating the patients with the pathways and with the current practice along all the observation period. The quality of the care will be assessed by monitoring the use of diagnostic and therapeutic procedures during hospital stay and by measuring key quality indicators at discharge.

**Discussion:**

This paper examines the design of the evaluation of a complex intervention. Since clinical pathways are made up of various interconnecting parts we have chosen the cluster-randomized controlled trial because is widely accepted as the most reliable method of determining effectiveness when measuring cost-effectiveness in real practice.

**Trial Registration:**

ClinicalTrials.gov ID [NCT00519038]

## Background

In Europe approximately 5% of all acute medical admissions relate to heart failure and in the United States heart failure is responsible for almost 1 million hospitalizations annually. Almost three quarters of these admissions are unplanned and worsening heart failure is responsible for half of these admissions [1,2].

The Acute Decompensated Heart Failure National Registry (ADHERE) showed that the hospital treatment of heart failure frequently does not follow published guidelines or conform to the Joint Commission on Accreditation of Healthcare Organizations (JCAHO) core performance measures, potentially contributing to the high morbidity, mortality and economic cost of this disorder [3,4]. ADHERE findings also suggested that the wide variations in conformity may reflect differences in training, guideline familiarity, and implementation of tools and systems to ensure that recommended care is provided and documented. Consequently the development of educational and quality improvement programs has the potential to considerably reduce the current variability in care, enhance guideline adherence, and improve outcomes for patients [5].

Clinical pathways has become a popular tool to achieve such goals [6-8]. Clinical pathways are a methodology for the mutual decision making and organization of care for a well-defined group of patients during a well-defined period with the aim to enhance the quality of care by improving patient outcomes, promoting patient safety, increasing patient satisfaction, and optimizing the use of resources. They are also developed by multi-professional teams [9]. Despite enthusiasm and diffusion, the widespread acceptance of clinical pathways remain questionable because very little prospective controlled data demonstrated their effectiveness [10-12].

The Experimental Prospective Study on the Effectiveness and Efficiency of the Implementation of Clinical Pathways was designed in order to conduct a rigorous evaluation of clinical pathways in hospital treatment of decompensated heart failure.

### Objectives

The primary objective of the trial was to evaluate the effectiveness of the implementation of clinical pathways for hospital treatment of heart failure among a sample of Italian hospitals. Our hypothesis was that the clinical pathways should be more effective than usual care in treating patients admitted in hospital for heart failure and that the clinical pathways should reduce patients' mortality during the stay and that they should improve patients' outcomes at discharge.

Secondary objectives were to estimate the efficiency and the appropriateness of the use of the resources associated with the intervention and its effects on other relevant outcomes.

The third objective was to define a statistical model able to predict in-hospital death and unscheduled re-admission.

A follow up study to evaluate the effectiveness of the intervention after three years from baseline was described in a separate protocol.

## Methods/Design

### The Project

The Experimental Prospective Study on the Effectiveness and Efficiency of the Implementation of Clinical Pathways was promoted and funded by the Italian Ministry of Health (Special Programs art. 12 bis D.lgs 229/99) and Marche Region. The study's Steering Committee defined the study's objectives, clinical topics, scheduling and design. The Regional Healthcare Agency of Marche Region coordinated and gave administrative support to the project and handled patients' data according to the Italian Data Protection act.

### Study design

We performed a cluster multi-centre randomized controlled clinical trial to evaluate the effect of applying clinical pathways to process and outcome indicators and to the costs sustained to assist the patients with heart failure. We compared the results obtained treating the patients with clinical pathways to the results obtained with the usual care. Since a clinical pathway is not a single intervention to be compared with a placebo but its eventual benefits come from a mix of complex actions that are implemented at the institutional level (appropriate use of practice guidelines and supplies of drugs and ancillary services, new organization and procedures, patient education, etc.), we randomly assigned hospitals, rather than individual patients, to either introduce the pathway or continue usual care [13-17].

**Figure 1:**
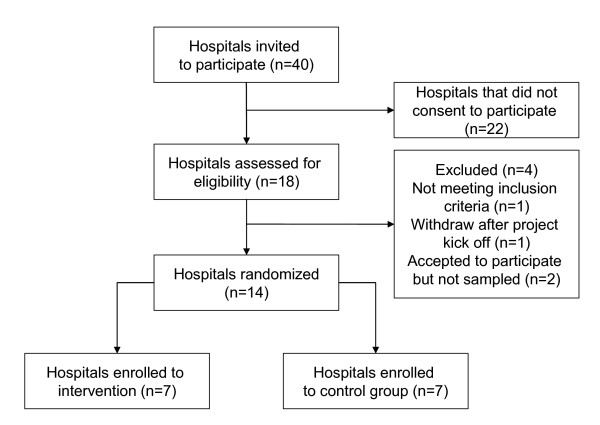
Flow diagram of the progress of the units through the trial.

Forty hospitals based in four Italian Regions were invited to participate to the study (Figure 1). Eighteen hospitals showed interest in the implementation of the clinical pathway for the hospital treatment of heart failure and were assessed for eligibility. Of the eighteen hospitals that asked to implement the clinical pathway for heart failure we selected and randomized fourteen community hospitals. We based the selection on the comparability of their location, patient population, facilities and teaching status (mean bed size of the hospitals assigned to clinical pathways was 245, in the usual care group was 262).

One hospital was excluded because it did not match with the inclusion criteria (it was a national institute specialized in geriatric hospital care and research) and one hospital withdrew after the project kick off meeting on a decision of the hospital management. To participate to the study the administrations of the hospitals had to be allow the institution to be allocated to either of the two strategies (clinical pathway or current practice) for a 1-year period and to agree not to implement a clinical pathway for the treatment of heart failure if assigned to the usual care group. Two hospitals could not assure do not implement a pathway if assigned to the control group and therefore were not sampled.

### Study evaluations

The primary outcome measure is in-hospital mortality. We will also analyze the impact of the clinical pathways comparing the length and the appropriateness of the stay, the rate of unscheduled readmissions, the customers' satisfaction and the costs treating the patients with the pathways and with the current practice along all the observation period. The quality of the care will be assessed by monitoring the use of diagnostic and therapeutic procedures during hospital stay and by the use of key quality indicators at discharge, as reported in previous studies [18-22]. The list of process and outcome indicators is reported in Table 1.

**Table 1:** The indicator set.

**Indicator (measure)**	**Typology**	**Criterion met/expected change**	**Measure**
In hospital mortality	Outcome	Differences in rates	%
Length of hospital stay	Outcome	Differences in mean values	days
Appropriateness of the stay (with Appropriateness Evaluation Protocol – AEP)	Outcome	Differences in rates	%
Costs of the stay (with Activity Based Costing – ABC)	Outcome	Differences in mean values	€ (euro)
Rate of unscheduled readmissions (within 31 days)	Outcome	Differences in rates	%
Patients' satisfaction (survey with 16 items questionnaire)	Outcome	Differences in mean values	score (1–10)
Diagnostic procedures during hospital stay	Process	Differences in rates	%
*Echocardiography*			
*Trans-oesophageal echocardiography*			
*Electrocardiography*			
*Chest x-rays*			
*Oximetry*			
*Weight monitoring*			
*Diuresis monitoring*			
Medical treatment during hospital stay	Process	Differences in rates	%
*Inotropes*			
*ACE-inhibitors*			
*Beta-blockers*			
*Diuretics*			
*Nitrates*			
*Other vasodilators*			
*Heparin*			
*Oral anticoagulants*			
*Anti-platelets agents*			
Left ventricular function (LVF) assessment rate at discharge (or planned for after discharge)	Process	Given to all patients	%
Rehabilitation rate at discharge (or planned for after discharge)	Process	Given to all patients	%
Advice/counseling rate for smoking cessation at discharge	Process	Given to all patients (current smokers)	%
Written instructions rate at discharge (activity level, diet, discharge medications, follow up, weight monitoring and what to do if symptoms worsen)	Process	Given to all patients	%
ACE-inhibitor rate at discharge (without contraindications, with LVF < 40%)	Process	Given to all patients	%

### Study sample

The sample included all the patients treated by the hospitals during the experimental period with a principal diagnosis of heart failure (all ICD-9CM codes included in 428.xx code). We calculated the sample size needed to detect a statistical difference in the mortality rate. Since in Italy the in-hospital mortality rates range from 5% to 17%, we expected that clinical pathways succeeded to control mortality to 5% to be clinically relevant [23-25]. Based on this goal a sample size of 424 patients (212 in each group) was required for the study to have 80% power at the 5% significance level (two-sided). The sample size calculation was performed according to standard criteria for cluster randomized trials. We adjusted the sample size using an inflation factor of 1.51 to account for the cluster randomization: 7 clusters per trial arm, cluster size of 30 patients, ICC of 0.018 [26-29].

At baseline we verified the comparability of the two groups at the admission measuring patients' age, sex, co-morbidities, risk factors and symptoms severity (Table 2). Patients with a current AMI or unstable angina were excluded from the study.

**Table 2:** Characteristics of 429 Hospital Patients in the Clinical Pathway and Usual Care Study Groups (demographics, risk factors and disease severity at admission).

**Variable**	**Clinical pathway **(n = 214)	**Usual Care **(n = 215)	**p value**
Male gender	102	110	0.50
Mean age in years (SD)	81.7 (8.3)	79.6 (8.5)	0.011
*Admitted from*			
General practitioner	106	109	0.77
Home	108	106	
*Severity at admission*			
NYHA II	16	15	
NYHA III	117	114	0.87
NYHA IV	81	86	
*Co morbidities*			
Hypertension	154	161	0.58
COPD	52	58	0.58
Diabetes	41	38	0.71
Smoking	34	31	0.68

### Intervention

The project started at each hospital with a ground round that showed the project protocol. Each hospital was assigned one methodological leader by the study Steering Committee (physicians or nurses with at least a two years experience with clinical pathways) that assisted local multidisciplinary teams in the development of the pathways and in the project implementation. The composition of each team was different in each institution and included general hospital-based physicians, cardiologists, epidemiologists, pathologists, psychologists, nurses, hospital-based pharmacists, social workers and administrative peoples. The teams were formed on a voluntary base, received a 3 days training in the development of clinical pathways and constructed the clinical pathways over a 6-month period.

The teams analyzed their care processes, did research for the best evidences and defined the appropriate goals to satisfy the multidimensional needs of the patients. These results were detailed into protocols and documentation, including the sequence of events and expected progress of the patients over time. The tasks for each professional were defined according to the following care categories: patients' evaluation; education of patients and families; planning of discharge; diagnostic exams; interventions and procedures; consultancies; medical treatments; nutrition; patients' safety [6,11].

The clinical pathways were analyzed by the EBM unit of the Regional Healthcare Agency of Marche and they were judged consistent with current recommendations for the diagnosis and the treatment of heart failure. After the validation of the pathways each team educated in its hospital the staff to the use of the clinical pathway and monitored the use of the pathway.

### Data analysis

Data were prospectively collected by local staff both in intervention and in control groups (physician and nurses who were trained in two pre-study educational events). We did not use incentives for the local staff.

The analysis will be performed by the research team. In addition to common descriptive statistics (Fisher exact and Kruskal Wallis test for categorical and continuous variables, respectively), that will be performed at the cluster level, the differences in the rate of in-hospital deaths and unscheduled admissions across groups and according to each variable under study will be evaluated using random-effects logistic regression, thus accounting for the clustering effect [30-33]. Variables will be included if significant at the 0.10 level (backward approach), with the exception of age which will be forced to entry. The presence of multicollinearity, interaction and higher power terms will be assessed to check final model validity. Patients who died during the study will be excluded from the regression model evaluating unscheduled readmissions because they could not be re-admitted.

Statistical significance will be defined as a two-sided p-value < 0.05. All analyses will be intention-to-treat and will be carried out using STATA statistical software, version 8.2 (Stata Corporation, College Station, Texas, 2003).

### Ethics

The project was exempt from ethical clearance according to the Italian Ministry of Health law number (ex art. 12bis D.lgs 229/99). Moreover the aim of the study is to improve quality of care through clinical pathways and thus should not imply any risk for the patients affected by the study. It is difficult to imagine that our intervention based on better evidences and appropriate use of technologies and drugs could worsen the quality of care when compared to usual care. So according to other experiences dealing with clinical pathways or implementation of evidence based guidelines in practice we think that a Committee of Research Ethic would not consider it necessary to submit the protocol for approval [34,35].

## Discussion

Even though randomized controlled trials are widely accepted as the most reliable method of determining effectiveness, clinical pathways has not been studied sufficiently in this way. Typically controlled trial design is not used in evaluating clinical pathways because the context level adaptation, which is essential for pathways to work, is perceived as inappropriate in the trial design, likely for the difficulty of keeping replicable and recognizable the intervention [36]. According to Hawe we think that a controlled trial design is appropriate to evaluate clinical pathways as well as other complex interventions and that it is possible to standardize the intervention (the clinical pathways) effectively. To this purpose we defined as standard the steps in the change process or the key functions that the elements of the intervention were meant to improve according to each context. Also the definition of the quality care indicators helped. The indicators were driven by the theory and concerned the functions provided by the key elements of the intervention that were based on expected adherence to the same evidences. We think that this strategy based on combining local change standards to the use of shared evidence based indicators kept the integrity of the intervention in each site.

Since clinical pathways are made up of various interconnecting parts we have chosen the cluster-randomized controlled trial design because is widely accepted as the most reliable method of determining effectiveness in Health Services Research [37]. As reported in sample size literature in cluster-randomized controlled trial we had difficulties in defining the sample size for the dual nature of the trial that focused both on individuals and clusters. The sample size calculation was based on the number of individuals needed, while the randomization process was based on clusters. Since each additional cluster represented a large proportionate increase in the study size (and in associated costs) it was necessary to find the proper balance between the need to increase the number of the clusters and its organizational costs. According to cluster design the sample size calculation needed to predict not only the expected effect size, but also the anticipated cluster size and ICC and this was difficult because the lack of published data on clinical pathways. However we think that the number of clusters and of individuals included in our sample assured the viability of the trial [27,29,37,38].

We had further difficulties related to current Italian healthcare information systems, above all in the method of documenting and collecting data from current sources (clinical records, paper based abstraction tools, etc.). We think that with a more comprehensive information structure for the clinical pathways, data collection would be easier even though the actual process did not affect the quality of the data but only reduced the efficiency of its collection.

## List of abbreviations

ADHERE: Acute Decompensated Heart Failure National Registry;

JCAHO: Joint Commission on Accreditation of Healthcare Organizations;

AEP: Appropriateness Evaluation Protocol;

ABC: Activity Based Costing;

LVF: Left ventricular function;

ICD-9CM: International Classification Diseases 9^th ^revision Clinical Modification;

ICC: Intra Cluster Correlation;

AMI: Acute Myocardial Infarction;

EBM: Evidence Based Medicine.

## Competing interests

The author(s) declare that they have no competing interests.

## Authors' contributions

MP conceived and developed this study and wrote the manuscript. SM assisted in the cluster creation, in defining the indicator set and contributed to the manuscript. AG helped to design the study and to the manuscript. FDS gave input to the project, overviewed all the steps of the study design and did the final review of the manuscript. All authors read and approved the final manuscript.

## Pre-publication history

The pre-publication history for this paper can be accessed here:


